# Establishing and validating of an laboratory information system‐based auto‐verification system for biochemical test results in cancer patients

**DOI:** 10.1002/jcla.22877

**Published:** 2019-03-06

**Authors:** Cuie Yan, Yujuan Zhang, Jia Li, Jia Gao, Chanjuan Cui, Chun Zhang, Guiyu Song, Mengyao Yu, Jianjun Mu, Feng Chen, Xiaohong Han, Wei Cui

**Affiliations:** ^1^ Department of Clinical Laboratory, National Cancer Center/National Clinical Research Center for Cancer/Cancer Hospital Chinese Academy of Medical Sciences and Peking Union Medical College Beijing China

**Keywords:** auto‐verification, biochemical test, cancer, historical extremum, laboratory information system

## Abstract

**Background:**

To establish and validate an laboratory information system (LIS)‐based auto‐verification (AV) system by using large amounts of biochemical test results in cancer patients.

**Methods:**

An algorithm of the AV process was designed for pre‐analysis, analysis, and post‐analysis. The limit range check was adjusted three times, while the delta check criteria were first replaced by the same patients’ historical extremum results. AV rules of 51 biochemical test items were tested by using data of 121 123 samples (6 177 273 tests) in 2016 that were manually reviewed through the simulative i‐Vertification software of Roche. The improved and optimal AV rules were programed into our LIS and validated by using 140 113 clinical specimens in 2018.

**Results:**

The AV passing rate for samples tested in our laboratory increased from 15.57% to the current overall passing rate of 49.70%. The passing rate of each item for rule 3 was between 71.16% and 99.91%. Different cancer groups had different passing rate, while the disease group of liver, gallbladder, and pancreas always had the lowest passing rate. A total of 9420 reports (6.72%) were not verified by AV but could be verified by MV in 2018, while there were no reports that were verified by AV but not by MV. The TAT of March 2018 decreased with increase in sample size compared with the same time in 2017.

**Conclusion:**

We have firstly established an LIS‐based AV system and implemented it in actual clinical care for cancer patients.

## 1 INTRODUCTION

With the increasing annual incidence of cancer,^1^ the number of patients admitted to cancer specialist hospitals has risen considerably, along with the demands for shorter report release turnaround time (TAT) from both the clinicians and patients. In addition to the complex pathophysiological changes induced by cancer, the common treatment modalities such as radiotherapy, chemotherapy, and surgery frequently result in impaired liver and kidney functions.^2^, ^3^ Therefore, biochemical tests for liver and kidney function are routinely performed for cancer patients, accounting for more than 50% of the total laboratory workload. The verification of these tests is a major post‐analytical process,^4^ and the accuracy and timely release of the results are crucial for medical decision‐making. In recent years, various auto‐verification (AV) systems have been incorporated into standard diagnosis. However, most of them need an intermediate software to be installed between the laboratory information system (LIS) and the specific instruments,^5^, ^6^, ^7^ and the data that are not transferred to this software cannot be automatically verified. We developed an LIS system to achieve real‐time AV and decrease the TAT. This is the first study to give a detailed report of the AV process according to the characteristics of cancer patients and evaluate the clinical benefits of the AV system.

## 2 MATERIALS AND METHODS

### 2.1 Test results of cancer patients

#### 2.1.1 Data sources and extraction

All data were obtained from National Cancer Center/National Clinical Research Center for Cancer/Cancer Hospital, Chinese Academy of Medical Sciences, and Peking Union Medical College, and included information on all inpatient, outpatient, and emergency room visits. The diagnosis for the different patients was entered into the hospital laboratory system (HIS) according to the International Classification of Diseases (ICD), 10th Revision. The HIS is updated daily by clinicians and contains information on date of diagnosis, cancer site, histology, dissemination, and other variables. The data sharing between HIS and LIS was instrumental in enabling the extraction of relevant diagnostic data.

The data verified as the final results from manual testing and stored in our LIS from January 1, 2016, to December 31, 2016, were searched using the following keywords: cancer OR neoplasm OR carcinoma OR tumor OR neoplasia. From the 193 563 patients who underwent complete biochemical examination (51 items, Table 1) in 2016, data of 121 123 patients (6 177 273 test items) were screened and included for establishing of our AV rules.

**Table 1:** Limit range for AV rules

Items	Critical value	Rule 1	Rule 2	Rule 3		Adjusted
K (mmol/L)	<2.5 or >6.0	3.5‐5.3	3.44‐5.11	3.0‐5.3		A
NA (mmol/L)	<125 or >155	137.0‐147.0	133‐145.2	130‐147		A
CL (mmol/L)		99.0‐110.0	94.8‐108.2	94‐110		B
TCO2 (mmol/L)		22‐29	20‐29	17‐29		D
ALT (U/L)		9.0‐50.0	7‐111	5‐200		F
AST (U/L)		15.0‐40.0	11‐97	5‐200		F
LDH (U/L)		120.0‐250.0	125‐377	100‐400	>HBDH	E
GGT (U/L)		7.0‐45.0 (Female)	9‐229	5‐229		D
GGT (U/L)		10.0‐60.0 (Male)				D
ALP (U/L)		45.0‐125.0	39‐222	10‐220		D
HBDH (U/L)		72‐182	98‐281	60‐350		E
CK (U/L)		50.0‐310.0	23‐451	23‐400	>CK‐MB	C
CK‐MB (U/L)		7‐25	7‐46	5_30		E
GLU (mmol/L)	<2.7 or >22.2	3.89‐6.38	4.22‐10.66	3.0‐11.1		G
TBA (μmol/L)		0‐10	0.3‐20.5	0‐20		B
TBIL (μmol/L)		2‐21	4.1‐35.2	1‐40		E
DIBL (μmol/L)		0‐5.1	2‐17.2	0‐20	<TBIL	C
IBIL (μmol/L)		0‐11.97			=TBIL‐DBIL	
Urea (mmol/L)	>36	3.6‐9.5	2.2‐9.5 (Male)	2‐ Upper limit of reference range *1.2		C
Urea (mmol/L)	>36	3.1‐8.8	2.2‐7.9 (Female)	2‐ Upper limit of reference range *1.2		C
CRE (μmol/L)	>530	41‐81	40‐91 (Female)	30‐ Upper limit of reference range *1.2		E
CRE (μmol/L)	>530	57.0‐111.0	40‐123 (Male)	30‐ Upper limit of reference range *1.2		E
URIC (μmol/L)		202.3‐416.5	123‐481	70‐600		H
CA (mmol/L)	<1.6 or >3.5	2.11‐2.52	1.94‐2.52	1.6‐3.0		A
PHOS (mmol/L)		0.85‐1.51	0.6‐1.51	0.3‐1.8		A
Mg (mmol/L)		0.75‐1.02	0.68‐1	0.5‐1.1		A
CHOL (mmol/L)		2.85‐5.69	2.66‐6.82	2.5‐8.0	>HDL‐C + LDL‐C	E
TG (mmol/L)		0.45‐1.69	0.53‐3.91	0.3‐9.0		A
HDL‐CHOL (mmol/L)		0.90‐1.45	0.59‐2.15	0.6‐3.0		C
LDL‐CHOL (mmol/L)		<3.34	1.34‐4.93	0.5‐5		B
B2MG (mg/L)		0.8‐2.2	1.2‐3.8	0.5‐4.25		E
IgA (g/L)		0.85‐4.9	0.93‐4.72	0‐6		E
IgG (g/L)		8.0‐17.0	6.34‐18.67	4_25		E
IgM (g/L)		0.50‐3.70	0.29‐2.42	0.2‐7		E
FE (μmol/L)		10.6‐36.7	3.2‐30.3	3.0‐35		B
TRansFE (mg/dL)		200‐400	133.7‐344.2	100‐450		E
TP (g/L)		65.0‐85.0	51‐80.9	40‐90		E
ALB (g/L)		40.0‐55.0	29.6‐50.3	20‐60		E
G (g/L)		20‐40			=TP‐ALB	
A/G		1.2‐2.4	1.01‐2.27	0.9‐2.6		E
PALB (mg/dL)		20‐40	8‐37	5‐45		E
Lpa (nmol/L)		0.0‐75.0	2.6‐232.8	0‐232		B
SOD (U/mL)		129.0‐216.0	109.3‐202	80‐300		E
HCY (μmol/L)		0‐20.0	4.1‐30.4	0‐35		C
CRP (mg/dL)		0.0‐0.6	0‐9.06	0‐10		C
ADA (U/L)		4.0‐24.0	5.3‐22.1	2‐40		E
APOA (g/L)		1.04‐2.02	0.7‐1.92	0.4‐4		E
APOB (g/L)		0.66‐1.33	0.5‐1.59	0.3‐3		E
ALB(SPE) (%)		52.0‐62.8	46.6‐66.4	40‐70	ALB(SPE) > γ‐G(SPE), The sum of the SPE items was equal to 100%	E
α1‐G(SPE) (%)		3.1‐4.6	2.8‐8.4	2‐8.4		B
α2‐G(SPE) (%)		7.0‐11.1	6.4‐14.4	5‐15		E
β1‐G(SPE) (%)		5.3‐7.8	4.6‐7.3	3‐12		E
β 2‐G(SPE) (%)		3.3‐6.4	3.3‐7.2	2‐9		E
γ‐G(SPE) (%)		13.1‐23.3	10.9‐24.8	10‐30		C

A: On the basis of rule 1 and rule 2, make adjustments by referring to our critical value.

B: Rules 1 and 2 were integrated.

C: On the basis of rule 1 and rule 2, the upper limit was adjusted according to the verification experience by senior technicians.

D: On the basis of rule 1 and rule 2, the lower limit was adjusted according to the verification experience by senior technicians.

E: On the basis of rule 1 and rule 2, the upper and lower limit were adjusted according to the verification experience by senior technicians.

F: Lower limit was referred to original reference range, and the upper limit was adjusted according to the verification experience by senior technicians.

G: The upper limit was adjusted according to the diagnostic criteria of diabetes.

H: The lower limit was adjusted according to the characteristics of patients in our hospital, and the upper limit was adjusted according to the definition of the increase of uric acid level in patients with renal insufficiency.

A/G, albumin/globin; ADA, adenosine deaminase; ALB(SPE), albumin (serum protein electrophoresis); ALB, albumin; ALP, alkaline phosphatase; ALT, alanine aminotransferase; APOA, apolipoprotein A; APOB, apolipoprotein B; AST, aspartate amino transferase; B2MG, β2 microglobulin; CA, calcium; CHOL, cholesterol; CK, creatine kinase; CK‐MB, creatine kinase isoenzyme; CL, chlorine; CRE, creatinine; CRP, C‐reactive protein; DIBL, direct bilirubin; FE, iron; G, globin; GGT, gamma‐glutamyl transferase; GLU, glucose; HBDH, alpha‐hydroxybutyric dehydrogenase; HCY, homocysteine; HDL‐CHOL, high density lipoprotein cholesterol; IBIL, indirect bilirubin; IgA, immunoglobulin A; IgG, immunoglobulin G; IgM, immunoglobulin M; K, kalium; LDH, lactic dehydrogenase; LDL‐CHOL, low density lipoprotein cholesterol; Lpa, lipoprotein a; Mg, magnesium; NA, sodium; PALB, pre‐albumin; PHOS, phosphorus; SOD, superoxide dismutase; TBA, total bile acid; TBIL, total bilirubin; TCO2, total carbon dioxide binding; TG, triglyceride; TP, total protein; TRansFE, transferrin; URIC, uric Acid; α1‐G(SPE), α1‐globulin (serum protein electrophoresis); α2‐G(SPE), α2‐globulin (serum protein electrophoresis); β1‐G(SPE), β1‐globulin (serum protein electrophoresis); β2‐G(SPE), β2‐globulin (serum protein electrophoresis); γ1‐G(SPE), γ1‐globulin (serum protein electrophoresis).

#### 2.1.2 Data classification

Combined with the clinical diagnosis of the patients, the data were divided into 11 cancer groups: A—lung, mediastinum, and thymus (n = 27,457), B—liver, gall bladder, and pancreas (n = 13,762), C—stomach, cardia, and esophagus (n = 13,942), D—the left, right, and both breasts (n = 16,071), E—cervix, ovary, uterus, vagina, and pelvic cavity (n = 10,490), F—colon, rectum, appendix, and small intestine (n = 11,400), G—nose, thyroid, parotid gland, oral cavity, tonsil, cheek, and brain (n = 10,995), H—kidney, bladder, prostate, and urethra (n = 3,388), I—lymphoma (n = 2,614), J—bones (n = 536), and K—other cancers (like teratocarcinoma; n = 10,450).

### 2.2 Instruments

All tests were performed using the automatic biochemical analyzers C701 and C702 (Roche, Germany), except for ALB (SPE), α1‐G (SPE), α 2‐G (SPE), β1‐G (SPE), β2‐G (SPE), and γ‐G (SPE), which performed using CapillaryS2FP (Sebia, France).

### 2.3 Construction of the AV algorithm

**Figure 1:**
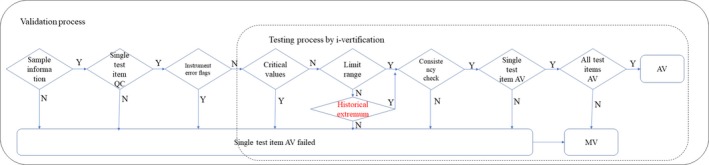
Algorithm design of AV

The AV algorithm was designed according to International organization for Standardization (ISO) 15189,^8^ College of American Pathologists (CAP) Checklist^9^ and Clinical and Laboratory Standards Institute (CLSI) AUTO‐10A^10^ (Figure 1), which covered the entire analytical process, that is, pre‐analysis (eg, patient diagnosis), analysis (eg, sample information, quality control, instrument status flags), and post‐analysis (eg, previous results). The limit range check was adjusted three times, first by using conservative reference range, and then using 95% confidence interval for each test item from the historical results in 2016, and finally adjusted by three technicians and three clinicians according to the individual cancer patients. The delta check was first replaced by the same patients’ historical extremum, and the critical value check and consistency check were also selected for the AV process. Qualified samples, quality control (QC) results, and properly performing instruments are the pre‐requisites for using the AV system. The order of validation using this system was critical value check, followed by limit range check, historical extremum, and finally consistency check for each single test item, and then for all test items. A sample passed the AV only if each single test item passed the process, while the results that failed any of the above rules were manually verified (MV).

### 2.4 Establishment of AV rules

The principles for establishing the AV rules were as followed:

#### 2.4.1 QC

Our laboratory routinely uses internal QC (IQC) and takes part in external quality assessment (EQA) of the National Center for Clinical Laboratories in China thrice a year. Daily IQC results were stored in LIS and were evaluated using Levey‐Jennings charts and Westgard quality control multi‐rules.

#### 2.4.2 Instrument error flags

The instruments were programed to give alerts for any problems with the reagents, barcodes, samples, or mechanical failure, for example, in the event of reagent crystallization or blood clotting. In addition, the results that were out of the analytical range also generated a warning flag and required sample dilution prior to re‐analysis.

#### 2.4.3 Critical value

The critical values used in our hospital were determined locally with clinicians and are listed in Table 1. Results that were outside the range of these values required verification by a technician, and those within the range were subjected to the limit range check.

#### 2.4.4 Limit range check

We first used conventional reference intervals as the limit range to verify the results (*rule 1*), then calculated the 95% confidence interval for each test item from the historical results of 2016 (*rule 2*), and at last adjusted each test item limit range with three technicians and three clinicians (*rule 3*; Table 1).

#### 2.4.5 Historical extremum

To the best of our knowledge, this is the first study to compare current test results with the same patients’ historical extremum results. Cancer patients need regular reviews and follow‐ups after treatment, and therefore, each patient has multiple biochemical test results which could be stored in our LIS and be compared. Historical extremum was the lowest and highest values within 1 year for one patient. For example, the lowest and highest ALT values for one patient were 5 U/L and 100 U/L, respectively, in 1 year, and then, his/her historical extremum was 5‐100 U/L. For one patient, his/her current test results can only be compared with his/her own historical extremum for rules 2 and 3.

#### 2.4.6 Consistency check

Due to the dynamic biological changes in acute illnesses, clinical test results often fluctuate, making it very difficult to perform a consistency rule check. A consistency check was established for only a small portion of the tests based on clinical and practical diagnostic criteria. For example, TC ≥ HDL‐C + LDL‐C, LDH > HBDH, CK > CK‐MB, TBIL > DIBL, IBIL = TBIL‐DBIL, and G = TP‐ALB et al.

All the test data were first imported into the AV simulation analysis platform i‐Vertification (provided by Roche company), and the best results limit ranges were confirmed by analyzing the AV passing rate. The LIS system engineer directly wrote programs into our LIS according to the AV rules (POWER LIS software, provided by the Beijing Hai Hui information technology co., LTD).

### 2.5 Validation method

Since the AV rules were based on historical data in 2016 and were written to our LIS system in 2017, they had to be validated with actual patient results before uploading, according to the CLSI Auto10‐A Guidelines. Therefore, we reanalyzed 140 113 clinical specimens in 2018 to verify whether rule 3 was able to meet our requirements. Since our laboratory does not have a pre‐processing system, the samples were verified manually to be without visible hemolysis, jaundice, lipidemia etc The system and rules ran well and did not demonstrate any error flags. TAT was defined as the time from the receipt of specimens in our laboratory to the time when the report was released to clinicians or patients.

## 3 RESULTS

### 3.1 The optimized rule 3 had the highest AV passing rate

**Figure 2:**
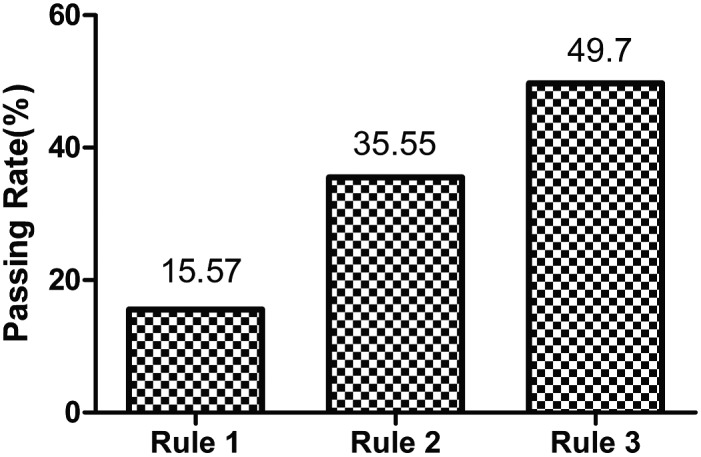
Passing rates for the three rules

To acquire the best AV rule, a total of 121 123 samples and 6 177 273 tests were collected and imported into the AV simulation analysis platform i‐Vertification. Rule 3 showed the highest AV passing rate of 49.70%, while rules 1 and 2 had respective passing rates of 15.57% and 35.55% (Figure 2). The passing rate of each item for the three rules is summarized in Table S1. The passing rate of each item for rule 1 ranged between 55.67% and 97.90%. After adjusting the limit range for each test item from the 2016 results as 95% confidence interval, the average passing rate of each item for rule 2 increased from 81.60% to 95.87%. The passing rate for the individual items (TCO2, AST, GGT, CK, GLU, DIBL, URIC, CHOL, TG, HDL‐CHOL, LDL‐CHOL, B2MG, IgG, IgM, FE, TRansFE, TP, ALB, PALB, Lpa, HCY, CRP, APOA, APOB, ALB [SPE], α1‐G [SPE], α2‐G [SPE], β1‐G [SPE], β2‐G [SPE], and γ‐G [SPE]) were improved compared to rule 1, with HDL‐CHOL showing the most significant improvement from 55.67% to 88.81%.

In addition, the lowest limit for LDH, HBDH, GLU, TBA, TBIL, DBIL, TG, B2MG, IgA, LPA, HCY, and ADA, and the highest limit for K, NA, CL, MG, IgA, IgM, FE, TRansFE, TP, ALB, A/G, PALB, SOD, ADA, APOA, and β1‐G (SPE) were within the reference range (Table 1), which indicated that rule 2 was not reasonable for these items. Therefore, we again adjusted the limit range for these items with our laboratory technicians and designed rule 3, which showed higher passing rate compared to rule 2. The average passing rate of each item for rule 3 was between 71.16% and 99.91%. Except for CK‐MB, IBIL, G, CRP, and α2‐G (SPE), the passing rate for all items improved compared to rule 2, with β1‐G (SPE) showing the greatest improvement (from 95.54% to 99.91%), and CRP showing a lower passing rate (from 92.32% to 71.16%).

### 3.2 Different cancer groups had different passing rate

The passing rates for each cancer group were 7.06%‐25.20%, 20.99%‐48.03%, and 29.97%‐65.50%, for rules 1, 2, and 3, respectively (Figure S1). Our results showed that with each adjustment, the passing rate was higher than before, while the disease group of liver, gallbladder, and pancreas (group B) always had the lowest passing rate.

### 3.3 Work efficiency analysis in actual patient results

The AV program based on our LIS could be directly applied to routine clinical work. The verified rule 3 showed a passing rate of 58.46% when used on 140 113 clinical specimens in 2018 (Table 2), which is consistent with the testing results. A total of 81 910 reports (58.46%) were verified by AV and MV, while 48 783 reports (34.82%) were not verified and required re‐evaluation or dilution. A total of 9420 reports (6.72%) were not verified by AV but could be verified by MV, while there were no reports that were verified by AV but not by MV. Therefore, the probability of releasing false results is none when the passing rate is increased.

**Table 2:** Comparison of MV and AV in 2018

Method	MV			
		Pass	No pass	Total
AV	Pass	81 910 (58.46%)	0 (0)	81 910
	No pass	9420 (6.72%)	48 783 (34.82%)	58 203
	Total	91 330	48 383	140 113

**Figure 3:**
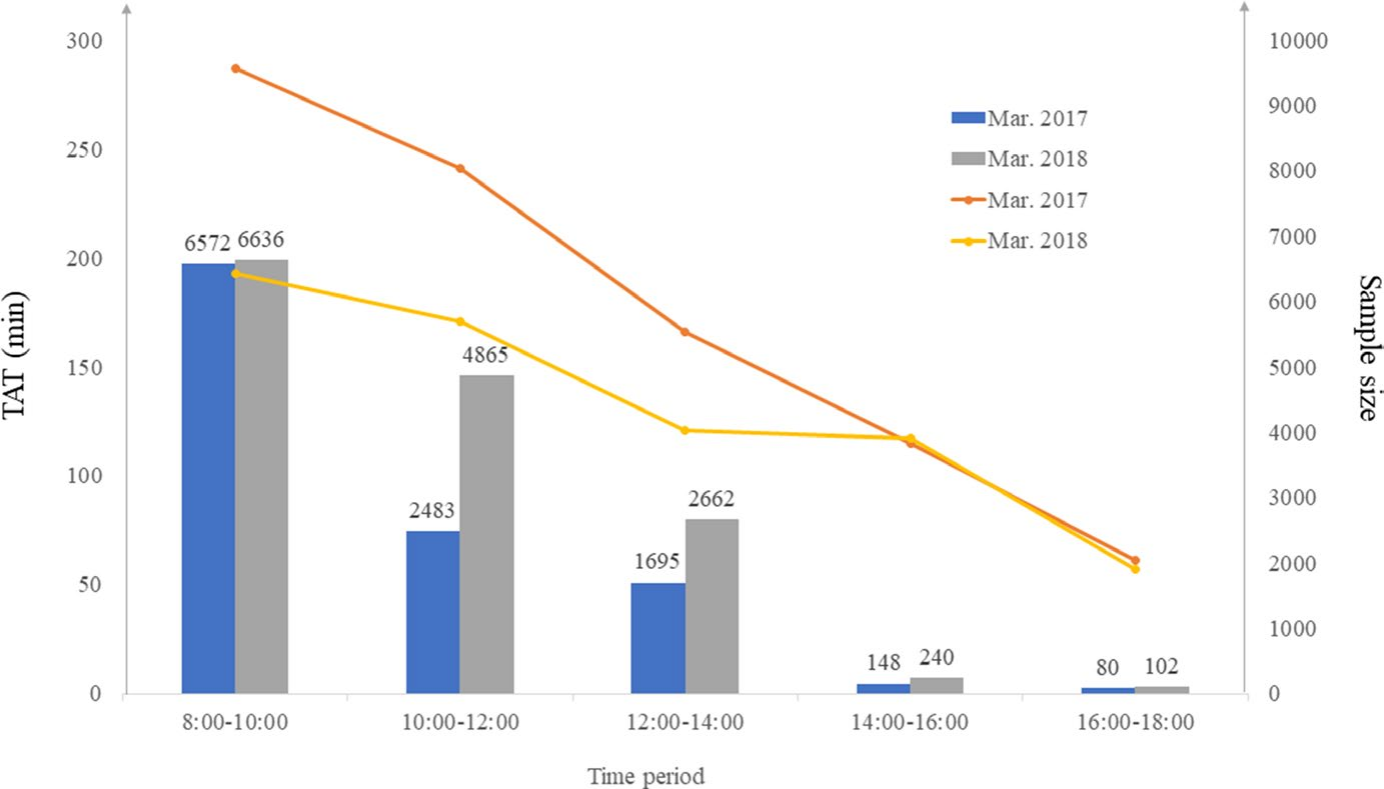
Work efficiency analysis

The TAT of 14 505 laboratory samples of March 2018 was compared with the same period last year (March 2017, n = 10 978) by different time period (Figure 3). The TAT decreased with increase in sample size. During the peak detection periods (8 am‐2 pm), the TAT was shortened by more than 1 hour. The time and labor expended by the laboratory staff were unaffected despite the increasing number of samples, as well as the need for manual validation of some samples.

## 4 DISCUSSION

Verification of clinical laboratory reports involves different technicians in the pre‐analytical, analytical, and post‐analytical phases, particularly in specialized cancer hospitals which run thousands of biochemical tests every day. AV of biochemical tests is a significant part of decision‐making and has benefitted from the development and application of artificial intelligence to the medical field in the last 20 years.^4^, ^11^ Although CLSI AUTO‐10A guideline has provided a general framework for AV, but it also advised each laboratory should design, implement, validate, and customize rules based on the needs of its own patient population. We previously found these rules were not applicable to cancer patients. Therefore, we summarized the manually validated results of our hospital, transformed it into computer language, and established the AV rules for the biochemical tests of cancer patients. Our LIS is programed to distinguish in real time whether the results need any further manual intervention for as long as the tests are carried out. This is the first study to give a detailed report of the LIS‐based AV process according to the clinical characteristics of cancer patients.

Since the limit range‐based criteria have the greatest impact on AV,^5^ we adjusted it three times to improve the AV passing rate. The reference range was first used, replaced with 95% confidence interval of each test item from the 2016 results, and finally adjusted by technicians according to their experience. This increased our passing rate from 15.57% to 49.7%, which is still lower compared to other studies. For example, the total AV rate for all thyroid function and sex hormone tests was 77.06% in a previous study,^6^ while Randell et al^5^ increased the AV rate of all clinical chemistry and immunoassay tests to >90% by using the Six Sigma approach. There were several possible reasons for our low passing rate. First, we had calculated the sample AV, the pre‐requisite for which was that each test item had to pass. Each biochemical sample in our study included 51 test items, which is far more than what others have tested. This might have lowered the total sample AV, although the passing rate of the individual items was not lower (>70%). Secondly, successful and continued use of AV requires investment in personnel and training over several years. The AV rate for tests performed in the core clinical chemistry laboratory has increased over the course of 13 years from 40% to the current overall rate of 99.5%.^7^ Therefore, AV is a continually evolving process, and we can still improve on the current rate.

The delta check criteria were first replaced by the same patients’ historical extremum results to accommodate their long‐term follow‐up. The powerful storage of our LIS allows the current test results of patients to be compared with his/her own test results within 1 year, which enables only patients’ physiological variations to be considered, and variations among individuals, the inherent flaw of delta check, should be avoided. Due to the huge sample size, the lowest and highest values for 51 test items were chosen in 2016 as the detection extremum range (Table S1).

In addition, the different cancer groups had different passing rates, and with each adjustment, the liver, gallbladder, and pancreas group always had the lowest passing rate. This could be due to the fact that tumors in these areas directly influence the biochemical tests. A qualified AV process should cover the entire analytical process, including patient diagnosis. However, accurate patient diagnosis is difficult, especially for the first time patients, and strongly dependent on information sharing between HIS and LIS. Therefore, we suggest that the best AV rules should be established by disease groups. Furthermore, since we wrote the algorithm of the AV process directly into LIS without an intermediate software,^12^, ^13^ the test results could be released directly to the physicians as soon as they passed the AV process.

Prior to uploading, our AV process was validated with 140 113 clinical specimens in 2018, and 6.72% of the reports which could be released directly were still intercepted. There is no analytical error yield based on our work. Due to the immediate release of AV results, the TAT of patient reports was shorter than those manually verified in the same period last year. In addition, the time and labor expended by the laboratory staff were unaffected despite the increasing number of samples, as well as the need for manual validation of some samples. Taken together, the ability to auto‐verify even a small percentage of the results can increase productivity and save labor.

Compared to the conventional manual verification which is tedious, time‐consuming, and (human) error‐prone, the biggest benefit of AV is the consistency of the test results. However, despite the advantages of AV, there are potential negatives. For example, the validation of AV was time‐consuming and required high attention to details. Furthermore, even the most thorough AV can miss unexpected instrument error flags or other rare events. It is also not possible to test every conceivable combination of rules. In conclusion, we have established and implemented an LIS‐based AV for the biochemical tests of cancer patients, although our AV passing rate of the rules still need to be improved compared to the commercially available AV software systems. With the development of information technology, report review will become more and more convenient and personalized.

## CONFLICT OF INTEREST

The authors have no conflicts of interests.
