# Supplementary figures and images for: Establishing and validating of an laboratory information system‐based auto‐verification system for biochemical test results in cancer patients
Source: J Clin Lab Anal. 2019 Mar 6;33(5):e22877. doi: 10.1002/jcla.22877 (PMC6595299; doi:10.1002/jcla.22877)

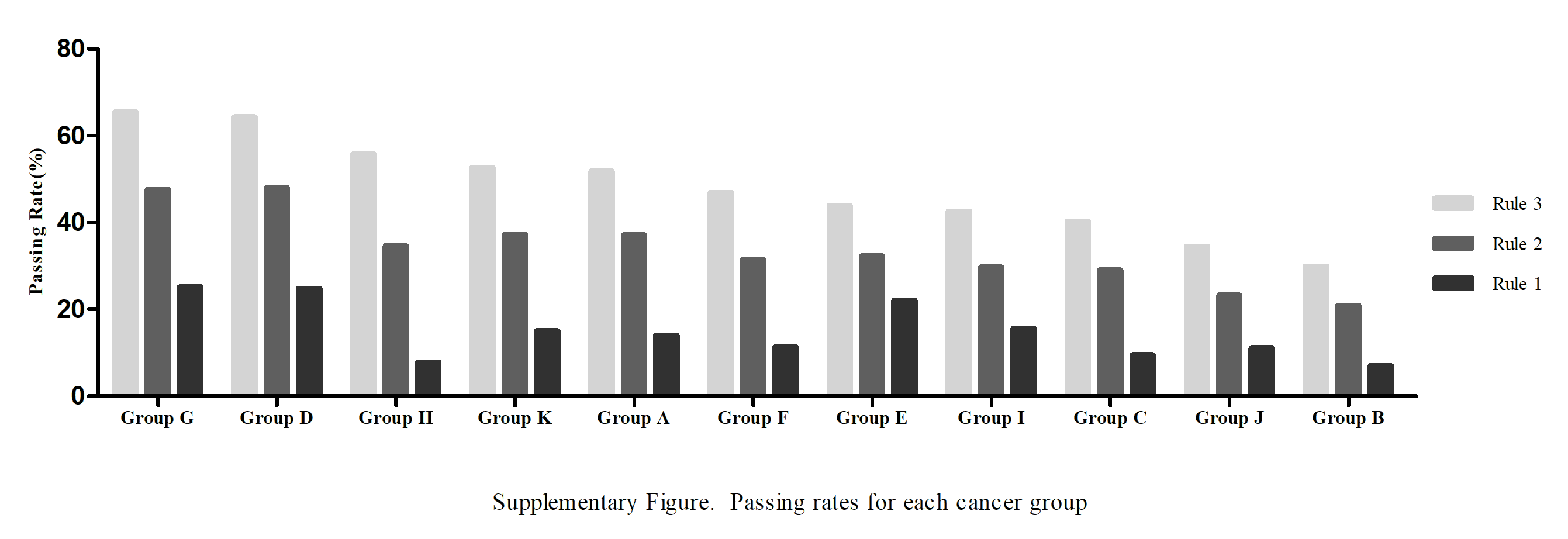

Supplement: Supplementary file 1 [file JCLA-33-e22877-s001.tif]
