# Supplementary material for: Establishing and validating of an laboratory information system‐based auto‐verification system for biochemical test results in cancer patients
Source: J Clin Lab Anal. 2019 Mar 6;33(5):e22877. doi: 10.1002/jcla.22877 (PMC6595299; doi:10.1002/jcla.22877)
Supplement: Supplementary file 2 [file JCLA-33-e22877-s002.docx]

Supplementary Table. Passing rate of each item for three rules

| Items | rule 1 (%) | rule 2 (%) | rule 3 (%) | extremum range |  |
| --- | --- | --- | --- | --- | --- |
| CRP | 70.94 | 92.32 | 71.16 | 0.08~76.9 μmol/L |  |
| ALP | 86.29 | 82 | 83.41 | 4~2937 U/L |  |
| A/G | 91.72 | 81.6 | 83.59 | 0.38~5.93 |  |
| K | 95.85 | 82.45 | 84.3 | 1.52~20.56 mmol/L |  |
| GLU | 80.24 | 82.8 | 84.32 | 0.36~48.58 mmol/L |  |
| Urea | 89.74 | 82.82 | 84.47 | 0.5~72 mmol/L |  |
| CL | 87.67 | 82.95 | 84.5 | 51.3~151.1 mmol/L |  |
| CRE | 87.09 | 82.89 | 84.52 | 10~1803 μmol/L |  |
| ALT | 86.2 | 83.25 | 84.82 | 1~6742 U/L |  |
| TBIL | 85.42 | 83.12 | 84.85 | 0.5~1006.8 μmol/L |  |
| DBIL | 70.77 | 83.7 | 85.21 | 0~722.1 μmol/L |  |
| NA | 88.52 | 83.21 | 85.51 | 73.4~182.1 mmol/L |  |
| AST | 79.33 | 84.06 | 85.57 | 1~15639 U/L |  |
| G | 93.46 | 85.66 | 85.66 | 4.9~84.4 g/L |  |
| PHOS | 87.69 | 84.02 | 85.89 | 0.11~5.49 mmol/L |  |
| ALB | 76 | 83.2 | 86.1 | 9.5~59.4 g/L |  |
| IBIL | 90.53 | 86.14 | 86.14 | 0~284.7 μmol/L |  |
| TP | 77.95 | 83.77 | 86.32 | 15.5~121.3 g/L |  |
| CK-MB | 90.97 | 88.36 | 86.91 | 1~3267 U/L |  |
| Mg | 90.8 | 85.22 | 87.62 | 0.24~2.4 mmol/L |  |
| CA | 86.31 | 85.01 | 87.81 | 0.91~4.64 mmol/L |  |
| CK | 79.16 | 87.32 | 87.85 | 3~9994 U/L |  |
| LDH | 89.22 | 86.29 | 88.09 | 54~22499 U/L |  |
| GGT | 82.66 | 87.12 | 88.88 | 1~3875 U/L |  |
| Lpa | 81.42 | 88.37 | 89.01 | 0.1~670 nmol/L |  |
| HBDH | 87.56 | 86.75 | 89.03 | 1~20703 U/L |  |
| TBA | 93.35 | 88.49 | 90.21 | 0~429.8 μmol/L |  |
| LDL-CHOL | 67.93 | 88.86 | 90.58 | 0.01~16.03 mmol/L |  |
| FE | 77.34 | 89.1 | 90.72 | 0.7~78.7 μmol/L |  |
| HDL-CHOL | 55.67 | 88.81 | 90.93 | 0.04~4.65 mmol/L |  |
| URIC | 78.73 | 88.14 | 91.11 | 15~1321 μmol/L |  |
| PALB | 74.79 | 88.76 | 91.22 | 1~164 mg/dL |  |
| ADA | 97.9 | 88.09 | 91.26 | 0.2~240.6 U/L |  |
| IgG | 85.14 | 88.22 | 91.28 | 0.05~66.76 g/L |  |
| CHOL | 82.56 | 89 | 91.33 | 0.53~34.54 mmol/L |  |
| IgA | 96.37 | 88.08 | 91.6 | 0.01~117.74 g/L |  |
| IgM | 86.38 | 88.15 | 91.62 | 0.01~648.74 g/L |  |
| TRansFE | 80.25 | 88.73 | 91.92 | 18.4~497.9 mg/dL | |
| SOD | 89.57 | 89.23 | 92.04 | 31~1174.1 U/mL | |
| HCY | 78.37 | 89.23 | 92.23 | 0.08~76.9 μmol/L | |
| B2MG | 72.86 | 91.09 | 92.55 | 0~15.6 mg/L | |
| TG | 71.96 | 88.62 | 92.89 | 0.02~16.64 mmol/L | |
| TCO2 | 88.27 | 91.19 | 92.93 | 3~49 mmol/L | |
| APOA | 83.71 | 89.8 | 93.0 | 0.01~3.45 g/L | |
| APOB | 73.78 | 89.87 | 93.78 | 0.01~4.97 g/L | |
| a1-G(SPE) | 68.94 | 95.76 | 94.41 | 0.9~24.3 % | |
| ALB(SPE) | 70.66 | 93.58 | 97.72 | 25~74.8 % | |
| a2-G(SPE) | 78.66 | 95.53 | 98.29 | 1.7~23.9 % | |
| γ-G(SPE) | 82.27 | 95.5 | 98.82 | 2.4~52.7 % | |
| ß2-G(SPE) | 90.74 | 95.87 | 99.61 | 1.7~30.6 % | |
| ß1-G(SPE) | 81.45 | 95.54 | 99.91 | 1.5~17.6 % | |
